# Correction to: Improvised use of a digital tool for social interaction in a Norwegian care facility during the COVID-19 pandemic: an exploratory study

**DOI:** 10.1186/s12913-022-07623-0

**Published:** 2022-02-17

**Authors:** Abeer Badawy, Mads Solberg, Aud Uhlen Obstfelder, Rigmor Einang Alnes

**Affiliations:** 1 Department of Health Sciences in Ålesund, Faculty of Medicine and Health Sciences, Norwegian University of Science and Technology, Larsgårdsvegen 2, 6009 Ålesund, Norway; 2 Center for Care Research, Department of Health Sciences in Gjøvik, Faculty of Medicine and Health Sciences, Norwegian University of Science and Technology, Teknologivegen 22, 2815 Gjøvik, Norway

**Correction to: BMC Health Serv Res 22, 136 (2022)**

**https://doi.org/10.1186/s12913-022-07526-0**

Following publication of this article [1], the authors identified two errors about the reference:

1. Reference 19 will have change in the authors order as follows:

Haldar M, Oppedal B, Askheim C, Rasmussen EB will be changed into Rasmussen EB, Askheim C, Oppedal B, Haldar M.

2. The first sentence of the last paragraph in the Background section should be changed from “To the best of our knowledge, there are only two studies (in Norwegian) that deal with experiences of using KOMP, a case study of elderly individuals with cancer [18], and the use of KOMP to bring people together and reduce loneliness among older adults [19].” to “To the best of our knowledge, there is a descriptive report (in Norwegian) that deals with experiences of elderly individuals with cancer who use KOMP to counter loneliness [18], and refined and theory-driven analysis of the use of KOMP to bring people together and reduce loneliness among older adults [19].”

The original article [1] has been corrected.
